# Antiedemic Effect of the Myosin Light Chain Kinase Inhibitor PIK7 in the Rat Model of Myocardial Ischemia Reperfusion Injury

**DOI:** 10.3390/cimb47010033

**Published:** 2025-01-06

**Authors:** Dmitry L. Sonin, Mikhail S. Medved, Asker Y. Khapchaev, Maria V. Sidorova, Marina E. Palkeeva, Olga A. Kazakova, Garry V. Papayan, Daniil A. Mochalov, Sarkis M. Minasyan, Ilya E. Anufriev, Daria V. Mukhametdinova, Natalia M. Paramonova, Ksenia M. Balabanova, Anastasia S. Lopatina, Ilia V. Aleksandrov, Natalya Yu. Semenova, Anna A. Kordyukova, Kirill V. Zaichenko, Vladimir P. Shirinsky, Michael M. Galagudza

**Affiliations:** 1 Institute of Experimental Medicine, Almazov National Medical Research Centre, 15B Parkhomenko Street, 194021 Saint Petersburg, Russia; medved_ms@almazovcentre.ru (M.S.M.); pgarry@mail.ru (G.V.P.); dan9ria@mail.ru (D.A.M.); ilya_anufriev_00@mail.ru (I.E.A.); natapa@bk.ru (N.M.P.); balabanova_km@almazovcentre.ru (K.M.B.); anasya.newcome@mail.ru (A.S.L.); aleksandrov_iv@almazovcentre.ru (I.V.A.); semenova_nyu@almazovcentre.ru (N.Y.S.); galagudza@almazovcentre.ru (M.M.G.); 2 Laboratory of Radio- and Optoelectronic Devices for Early Diagnostics of Living Systems Pathologies, The Institute for Analytical Instrumentation, Russian Academy of Sciences, 31-33A Ivana Chernykh Street, 198095 Saint Petersburg, Russia; annygm00@mail.ru (A.A.K.); kvz24@mail.ru (K.V.Z.); 3 Institute of Experimental Cardiology Named after Academician V.N. Smirnov, National Medical Research Centre of Cardiology Named after Academician E.I. Chazov, 121552 Moscow, Russia; askerkhapcha@gmail.com (A.Y.K.); mvs.peptide@gmail.com (M.V.S.); mpalkeeva@mail.ru (M.E.P.); olga-shadow@mail.ru (O.A.K.); shirinsky@gmail.com (V.P.S.); 4 Scientific and Educational Institute of Biomedicine, Pavlov First Saint Petersburg State Medical University, 6–8 Lev Tolstoy Street, 197022 Saint Petersburg, Russia; 5 Infochemistry Scientific Center, ITMO University, Lomonosova Str. 9, 191002 Saint-Petersburg, Russia; 6 Department of Pathophysiology with Clinical Pathophysiology Course, Pavlov First Saint Petersburg State Medical University, 6–8 Lev Tolstoy Street, 197022 Saint Petersburg, Russia

**Keywords:** myosin light chain kinase, peptide inhibitor PIK7, ischemic and reperfusion injury, myocardial infarction, no-reflow, microvascular hyperpermeability

## Abstract

Myocardial ischemia-reperfusion injury increases myocardial microvascular permeability, leading to enhanced microvascular filtration and interstitial fluid accumulation that is associated with greater microvascular obstruction and inadequate myocardial perfusion. A burst of reactive oxygen species and inflammatory mediators during reperfusion causes myosin light chain kinase (MLCK)-dependent endothelial hyperpermeability, which is considered a preventable cause of reperfusion injury. In the present study, a single intravenous injection of MLCK peptide inhibitor PIK7 (2.5 mg/kg or 40 mg/kg) was found to suppress the vascular hyperpermeability caused by ischemia/reperfusion injury in an in vivo rat model. The antiedemic effect of PIK7 is transient and ceases within 90 min of reperfusion. The early no-reflow detected for the first time after 30 min ischemia in this model of myocardial infarction reduces the area accessible for PIK7. Electron microscopy has shown membrane-bound blebs of endotheliocytes, which partially or completely obturate the capillary lumen, and few capillaries with signs of intercellular gap formation in samples obtained from the center of the early no-reflow zone in control and PIK7-injected rats. Co-injection of PIK7 with NO donor sodium nitroprusside (SNP) increases blood flow in the zone of early no-reflow, while reducing the increased vascular permeability caused by SNP.

## 1. Introduction

Early reperfusion is the main goal in the treatment of acute coronary syndrome. Timely reperfusion may prevent the death of ischemic myocytes and reduces microcirculatory damage and manifestations of the no-reflow phenomenon [1,2,3,4]. The no-reflow phenomenon is defined as the absence of complete myocardial perfusion at the level of the microcirculatory bed after elimination of the cause of coronary artery occlusion [4,5,6,7]. Depending on the method of diagnosis, the incidence of no-reflow phenomenon in patients with STEMI is up to 67% according to different sources [4]. The review by Konijnenberg (2020) proposed a new collective term, “coronary microvascular dysfunction”, which combines two main mechanisms of diminished myocardial perfusion in the microcirculatory bed in patients with STEMI after primary percutaneous coronary intervention: manifestations of ischemia/reperfusion injury of the coronary microvasculature and embolization of the distal coronary artery by thrombus fragments and atherosclerotic plaque components [6].

The pathophysiology of the no-reflow phenomenon recognizes the existence of two variants of the phenomenon: early and late. The distinction is based on the severity of ischemia. Early (primary) no-reflow is caused by a prolonged period of myocardial ischemia with a low level of residual blood flow (subendocardial and intramural layers). Secondary no-reflow is caused by ischemia-reperfusion injury and is manifested by a progressive deterioration of myocardial perfusion in areas of restored blood flow [1,8,9]. The main mechanism of primary and secondary no-reflow is endotheliocyte edema, which occurs at the stage of myocardial ischemia and worsens with the restoration of blood flow [9,10]. Electron microscopic evidence suggests that intracellular swelling of cardiomyocytes and endotheliocytes occurs during ischemia and results in the protrusion of cell membranes [9,11,12]. Although endotheliocytes are more resistant to ischemic injury, prolonged ischemia leads to swelling endotheliocytes with the formation of membrane blebs (membrane-bound bodies), whose size depends on the duration of ischemia. With prolonged ischemia, intracellular edema leads to complete occlusion of the microvascular lumen and to the appearance of primary no-reflow, the pathomorphologic sign of which is coagulation necrosis [1,9]. In the case of moderate damage to endotheliocytes, reperfusion injury additionally increases edema of endotheliocytes, cardiomyocytes, and intercellular space, leading to the extravascular compression of microvessels and the occlusion of their lumen with the formation of blood stasis and delayed no-reflow [4,7].

During ischemia, conditions of microvascular leakage are established; therefore, intercellular edema forms from the moment of restoration of blood flow. Ischemia and reactive oxygen species, as well as histamine, thrombin, and other active substances produced during reperfusion, activate myosin light chain kinase (MLCK), which leads to endothelial contraction, disruption of intercellular contacts, and a sharp increase in vascular permeability [13,14]. This results in myocardial edema and hemorrhage [4,5,6,7,15]. According to literature data, mice with a genetic knockout of 210 kDa MLCK as well as wild-type mice treated with a small-molecule MLCK inhibitor are more resistant to lung injury in experimental models of sepsis due to the preservation of microvascular endothelial barrier function [16]. These data suggest that pharmacological inactivation of MLC may be beneficial for the attenuation of cardiac reperfusion injury.

A promising antiedematous drug for adjuvant therapy of myocardial infarction is a novel proteolytically stable peptide inhibitor of MLCK, PIK7, designed at the National Medical Research Center of Cardiology, named after Academician E.I. Chazov, Ministry of Health of the Russian Federation [17]. Given the known role of increased vascular permeability in reperfusion injury and the possible benefits of MLCK inhibition [18], we investigated the effect of PIK7 on endothelial barrier function in vitro and coronary vascular permeability and efficacy in the limiting of no-reflow phenomenon in an in vivo model of ischemia and reperfusion of rat myocardium.

The PIK7 design is based on the structure of MLC11-19, a competitive peptide inhibitor of MLCK [19] capable of penetrating living cells [20]. Peptides composed of natural L-amino acid residues are generally highly susceptible to degradation by peptidases in blood plasma, and MLC11-19 is not an exception. Using 1H-NMR spectroscopy to study MLC11-19 degradation time course in human plasma in vitro, we observed that the peptide degradation starts at the N-terminus, yielding a peptide half-life of 0.5 h. Based on these data, the substitution of the second L-lysine residue in MLC11-19 for the D-lysine residue was undertaken, which produced a novel peptide, PIK7, with a half-life in plasma exceeding 5 h [17]. PIK7 was synthesized using a solid-phase peptide synthesis method utilizing the Fmoc-technology and was purified to >97% by HPLC [17].

The aim of the present study was to evaluate the effect of PIK7 on the vascular permeability in the no-reflow zone in a rat model of myocardial ischemia-reperfusion injury. Thioflavin S was used to map the boundaries of the no-reflow zone, and a modified Miles test using indocyanine green [21] instead of Evans blue was used to assess the severity of endothelial disruption in the risk zone.

## 2. Materials and Methods

### 2.1. In Vitro: Endothelial Barrier Function Assay

The functional effects of PIK7 were analyzed in cultured EA.hy926 human endothelial cells (ATCC, Manassas, VA, USA) in vitro by measurement of the thrombin-induced 70 kDa FITC-dextran permeability across cell monolayers grown on porous filters, as described in [22]. In separate experiments, quantitative calculation of the thrombin-induced phosphorylation of myosin regulatory light chain (RLC), both at Ser19 and Thr18/Ser19 in EA.hy926 cells pretreated with PIK7, was carried out using a Western-blotting-based method and isolated in vitro phosphorylated RLC as an external calibration standard, as reported previously [23]. Although MLCK does not diphosphorylate myosin RLC in cells, it cooperates with the protein kinase ROCK that uses MLCK-produced monophosphorylated RLC as a substrate for further diphosphorylation [22]. From this standpoint, it is important to include diphospho-RLC data into the account of PIK7 inhibitory activity in EA.hy926 cells.

### 2.2. In Vivo: Modeling of Myocardial Ischemia and Reperfusion

The study was performed on male Wistar rats of the SPF category (age 16–20 weeks, weight 350–400 g, Novosibirsk nursery) in accordance with the protocol approved by the Commission for the Care and Use of Laboratory Animals of the Almazov National Medical Research Centre (PZ_23_6_Sonin_DL_V3, 06.14.2023).

The study used an in vivo model of regional ischemia-reperfusion of the rat myocardium. Rats were anesthetized with isoflurane gas anesthesia, with constant monitoring and maintenance of body temperature at 37 ± 0.5 °C (ATC1000-220, World Precision Instruments, Inc., Sarasota, FL, USA). Rats underwent tracheostomy and artificial lung ventilation (CWE-SAR-830/AP, CWE Inc., Ardmore, PA, USA) with a gas mixture with 60% oxygen (respiratory rate—60/min, respiratory volume—3 mL/100 g body weight). Artificial ventilation was regulated by repeated analyses of arterial blood gases throughout the experiment (i-STAT^®^ System, Abbott Point of Care Inc., Princeton, NJ, USA). Arterial blood was taken from the right common carotid artery through a catheter (PE-50 Intramedic™ PE Tubing, Becton-Dickinson, Sparks, NV, USA) connected to a sensor (TruWave pressure transducer, Edwards Lifesciences LLC, Irvine, CA, USA) for measuring blood pressure (BP) and heart rate (HR) using software PhysExp X4 (Cardioprotect LLC, St. Petersburg, Russia). Femoral vein catheterization was performed for infusion of the tested solutions and dyes.

The chest was opened by an incision in the fourth intercostal space to induce regional ischemic reperfusion injury. The ribs were bred to expose the heart, then the pericardium was opened and a 6.0 polypropylene ligature with an atraumatic needle was inserted under the main branch of the left coronary artery, approximately 2 mm from its beginning [24]. The ends of the ligature were passed through an occluder—a small polyethylene tube ~7–8 cm (PE-90, Intramedic™ PE Tubing, Becton-Dickinson, Sparks, NV, USA)—and brought out. Myocardial ischemia was initiated after the end of surgical procedures and a 30 min stabilization period. Reversible myocardial ischemia was created by shifting the occluder down the ligatures and applying a surgical clamp to the occluder to prevent it from shifting backwards. Hemodynamic parameters were recorded immediately before the 30 min occlusion, 5 and 15 min after occlusion, at the beginning of reperfusion (at the 5th minute) and then every 30 min until the end of the experiment.

A solution of indocyanine green (ICG) at a dose of 1 mg/kg (Pulsion Medical Systems AG, Munich, Germany) was injected in rats intravenously for assessing the severity of endothelial disruption in the myocardial infarction zone in the first minutes of reperfusion (ICG-0′) or at the 90th minute of reperfusion (ICG-90′). ICG was dissolved in distilled water, then NaCl was added to the ICG solution to obtain a NaCl concentration of 0.9% and a final ICG concentration of 2 mg/mL and administered in 1 min in a volume of 0.5 mL.

A solution of 4% Thioflavin S (ThS) in 0.9% NaCl solution in a volume of 0.7 mL was bolus injected at the end of the 2 h reperfusion period, 15 s before heart excision to measure the size of the no-reflow zone.

### 2.3. Pharmacological Substances

PIK7 and sodium nitroprusside (Naniprus, Sopharma AD, Sofia, Bulgaria) were diluted in 0.9% NaCl solution immediately before injection and injected intravenously separately (without mixing with each other). The basic dose of PIK7 2.5 mg/kg used in this study was comparable to PIK7 concentration effective in experiments with cultured endothelial cells—100 uM [17]. Given the volume of circulating blood in rats, 55–70 mL/kg, we estimated a peak concentration of PIK7 in blood following its bolus i/v administration as approximately 50 uM. Additionally, a higher dose of PIK7 was used (40 mg/kg) to probe the dose–efficacy relationship. Based on previous rodent data, this higher dose of PIK7 was non-toxic [17].

The rate of intravenous injection of sodium nitroprusside was regulated by the blood pressure limit, so that during its injection it was in the range of 40–50 mmHg [25].

Exclusion criteria: SAD < 50 mmHg and/or heart rate < 300 at any time during the experiment.

**Figure 1:**
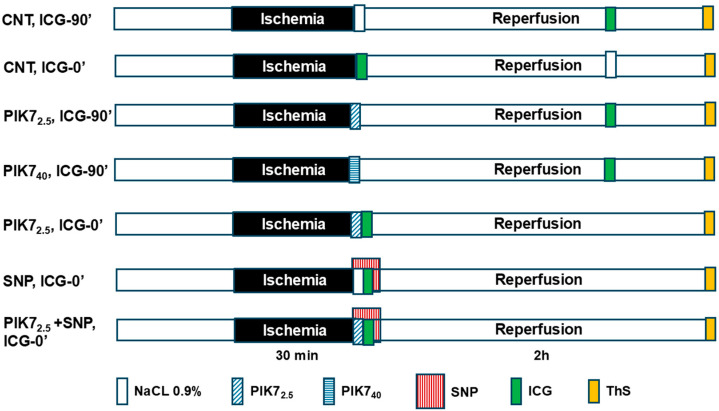
Protocol of the experimental study.

Figure 1 shows the protocol of the experimental study.

1. In the «CNT, ICG-90′» group, 30 min ischemia and 120 min reperfusion were created; a bolus of ICG solution was injected intravenously for 1 min at the 90th minute of reperfusion. Bolus ThS was intravenously injected 10 s before the excision of the heart (*n* = 9).
2. The protocol of the «CNT, ICG-0′» group is identical to the protocol of the «CNT, ICG-90′» group, except that the ICG solution was intravenously injected during 1 min of reperfusion (*n* = 7).
3. The protocol of the «PIK7 2.5, ICG-90′» group is identical to the protocol of the «CNT, ICG-90′» group, except that, 30 s before the end of ischemia, an intravenous solution of PIK7 was injected at a dose of 2.5 mg/kg for 1 min (*n* = 7).
4. The protocol of the «PIK7 40, ICG-90′» group is identical to the protocol of the «PIK7 2.5, ICG90′» group, except that PIK7 was injected at a dose of 40 mg/kg (*n* = 7).
5. The protocol of the «PIK7 2.5, ICG-0′» group is identical to the protocol of the «PIK7 2.5, ICG-90′» group, except that ICG was injected in the first minute of reperfusion immediately after the injection of PIK7 (*n* = 3).
6. The protocol of the «SNP, ICG-0′» group is identical to the protocol of the «CNT, ICG0’» group, except that intravenous injection of sodium nitroprusside solution began 30 s before the end of ischemia, and the ICG solution was injected into another femoral vein within 1 min of reperfusion (*n* = 6).
7. The protocol of the «PIK7 2.5 + SNP, ICG-0′» group is identical to the protocol of the «CNT, ICG-0′» group, except that intravenous injection of two solutions was started 30 s before the end of ischemia: sodium nitroprusside at a dose of 60 µg/kg and PIK7 at a dose of 2.5 mg/kg, followed by injection of ICG (*n* = 10).

### 2.4. Visualization of Ischemic Reperfusion Injury Ex Vivo

The left coronary artery was re-occluded at the end of the experiment (after 2 or 120 min of reperfusion), after which 2.5 mL of 2.0% Evans Blue (MP Biomedicals, Santa Ana, CA, USA) was injected through the femoral vein to identify the risk zone. The hearts were cut out and cut into five slices, 2 mm thick, parallel to the atrioventricular sulcus. The basal surface of each slice was photographed using a digital camera. The slices were immersed in a 1% solution of 2,3,5-triphenyltetrazolium chloride (TTC, ICN Pharmaceuticals, Costa Mesa, CA, USA) at a temperature of 37 °C (pH 7.4) for 15 min and photographed again to identify the infarction area and register the fluorescence area and ICG intensity. The images were analyzed using ImageJ (https://imagej.net/ij/, accessed on 28 August 2023). The risk area (Evans-negative area) was expressed as a percentage of the entire section, and the area of the myocardial necrosis zone (TTC-negative areas) was expressed as a percentage of the risk area. The values of the risk area and the infarction area for each heart were obtained by summing the data for the slices and calculating the average values.

### 2.5. Methods of Fluorescence Registration

Registration of ICG-fluorescence in the near-infrared range (820–900 nm) under excitation with radiation of 780–810 nm was carried out using a specially developed optical imaging system for the near-infrared fluorescence range [26]. It includes a diode laser with a wavelength of 808 nm, providing excitation of infrared fluorescence, and a multispectral video system.

A tube illuminator was used for visualizing ThS-fluorescence, in which a short-arc mercury lamp, HXP 120VIS Osram, with a power of 120 watts, was used as a light source. The radiation was selected by switchable light filters (in this case, a bandpass filter with a central wavelength of 390 nm, a width of 40 nm, FF01-390/40-25) and a liquid light guide for radiation delivery. Image registration was carried out using a multispectral shooting system based on a highly sensitive RGB television matrix.

### 2.6. Comparison of ICG- and ThS-Fluorescence Intensity in the Zone of Myocardial Ischemia/Reperfusion Injury

The obtained images with ICG- and ThS-fluorescence were evaluated using Image Pro Plus programs. To study the nature of the intensity of ICG- and ThS-fluorescence, a grid was drawn on the images of the risk zone of the second and third slices from the apex of the heart, the number of sectors and cells of which were the same and tied to the boundaries of the risk zone. The grid formed in this way was then transferred to images of slices stained with TTC, and with ICG- and ThS-fluorescence.

The grid allows comparison of the intensity of ICG- and ThS-fluorescence in the same section, as well as to find the dependence of the boundaries of necrosis zones from the edema zones at the beginning of reperfusion. The area of interest (ROI) was allocated in each cell in the area at risk (AAR) and in each cell of the reference sector. Then, within the selected ROI, the average value of the fluorescence intensity was calculated, which was expressed in conventional units (a.u.). To compare the fluorescence intensity between any cell in the AAR with an intact zone, the corresponding cell in the reference sector in the interventricular septum equidistant from the boundary sectors of the AAR was selected.

The quantitative comparison was carried out using the contrast parameter, subtracted by the following formula, contrast = (ROIaar − ROIref) ÷ ROIref, where ROIaar is the average value of the fluorescence intensity in a cell in AAR and ROIref is the average value of the fluorescence intensity in a cell in the reference zone. Before measuring contrast, the average value of background fluorescence measured at five randomly selected points around the measured slice was subtracted from the average values of fluorescence intensity [21].

### 2.7. Comparison of No-Reflow Zone Sizes Based on ICG and ThS Fluorescent Images

To calculate the no-reflow zones, planimetric imaging of cardiac sections was performed in the ImageJ program. For measurement of the area of the no-reflow zone, boundaries were outlined in ICG- and ThS-fluorescence images. The boundaries of the no-reflow zones were delineated by the peaks of ICG- or ThS-fluorescence, within which the outer zone with the maximum level of microvascular leakage and the inner, non-fluorescent region are located.

### 2.8. UHR ECG Registration and Signal Processing

The original method of reliable electrocardiographic control of ischemia appearance in investigations with experimental animals was used for continuous electrocardiogram (ECG) monitoring and control of ischemia and reperfusion onset [27,28]. The electrodes of conventional ECG (Cardiotechnica-ECG-8; Incart Ltd., St Petersburg, Russia) and ultra-high-resolution electrocardiography (UHR ECG) were implanted subcutaneously according to a standard scheme. The ECG UHR method aims to study the fine structure of electrocardiac signals and reveal additional meaningful diagnostic information for the early detection of electrophysiologic changes in the heart caused by ischemia.

### 2.9. Myocardial Histology

The heart samples were fixed in 10% buffered formalin for 48 h. Next, the material was dehydrated and impregnated with paraffin according to the generally accepted standard procedure. For histological analysis, sections with a thickness of no more than 5 microns were made, followed by trichromic staining using the Mallory method (BioVitrum, St Petersburg, Russia). The morphometry of 5 µm sections was used to quantify stasis regions (areas occupied by erythrocytes) using a Nikon Eclipse Ni-U optical microscope (Nikon Corporation, Tokyo, Japan) at 200× magnification and the NIS-Elements BR 4.3 computer program (Nikon Corporation, Tokyo, Japan). Stasis areas (target area) in the intramural layer of the risk area (total area 3 mm^2^) were detected using the ImageJ Color threshold tool. The percentage of the target area was calculated using the following formula: (target area/total area) × 100%.

### 2.10. Transmission Electron Microscopy

For electron microscopic examination, pieces of tissue with a thickness of 1.0–1.5 mm were prefixed in a mixture of 0.5% glutaraldehyde and 4% paraformaldehyde cooled to 4 °C, diluted with 0.1 M cacodilate buffer (pH 7.2–7.4). This was fixed in a 1% solution of osmium tetrachloride (all reagents are Sigma-Aldrich, St. Louis, MO, USA) cooled to 4 °C after 1.5–2.0 h. The material was dehydrated in solutions of ethyl alcohol of ascending concentration and absolute acetone. During dehydration, the tissue was contrasted with a 3.5% solution of uranyl acetate in 70% ethanol. Impregnation and filling with a mixture of araldites (Honeywell Fluka™, Buchs, Switzerland) with sample orientation was performed under a magnifying glass. Polymerization was carried out in a thermostat at 37 °C and 60 °C over the course of 3 days. Ultrathin sections (50–60 nm) were prepared using LKB-III ultratome (LKB, Stockholm, Sweden). The registration of changes in the structure of tissues and their photofixation were carried out on a transmission electron microscope FEI Tecnai G2 Spitit BioTWIN (FEI Company, Eindhoven, The Netherlands) at an accelerating voltage of 80 kV, provided by the Center for Collective Use of the Sechenov Institute of Evolutionary Physiology and Biochemistry of the RAS and on the transmission electron microscope HITACHI 7800 (Hitachi High-Tech, Hitachinaka, Japan), provided by the Center for Preclinical and Translational Research of the Almazov National Medical Research Center.

### 2.11. Statistical Analysis

The median of both the 25th and 75th percentiles [Me (25; 75%)] was used to describe features with a different distribution from the normal one. The Wilcoxon paired *T*-test was used to assess the reliability of differences before and after exposure. The U criterion (Wilcoxon–Mann–Whitney criterion) was used to assess the reliability of differences between two conjugate aggregates. Statistical data processing was carried out using the GraphPad Prizm 6 program.

## 3. Results

### 3.1. Effects of PIK7 on Endothelial Barrier Function and Myosin RLC Phosphorylation in Endothelial Cells In Vitro

**Figure 2:**
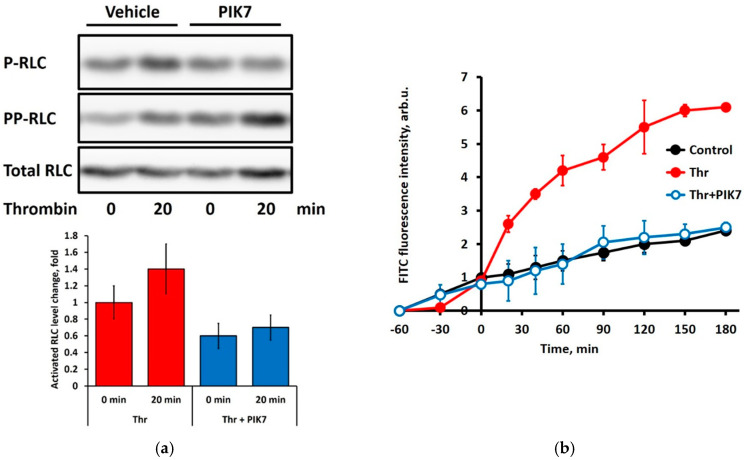
Effects of 100 µM PIK7 on the thrombin-induced EA.hy926 endothelial monolayer hyperpermeability and myosin RLC phosphorylation in EA.hy926 endothelial cells. (**a**) The levels of the thrombin-induced myosin regulatory light chain (RLC) phosphorylation in EA.hy926 cells were calculated based on sequential visualization of myosin RLC monophosphorylated at Ser19 (P-RLC), diphosphorylated at Thr18/Ser19 (PP-RLC), and total RLC on Western blots of endothelial cell samples using an external standard mixture containing RLC/P-RLC/PP-RLC, as described in [23]. Representative Western blots are shown. Bars below represent the sum of mono- and di-phosphorylated (activated) myosin RLC before (0 min) or 20 min after thrombin (Thr) administration normalized by the total RLC content in each sample. Data are presented as means ± SD, *n* = 4. (**b**) Effect of PIK7 on the thrombin-stimulated 70 kDa FITC-dextran permeability across the EA.hy926 endothelial cell monolayer. Cultured cells were preincubated or not with PIK7 for 60 min before 100 nM thrombin was added at 0 min. Cells in control were left untreated by PIK7 or thrombin. Data are presented as means ± SD, *n* = 5. *p* < 0.05 for all PIK7 data points within the 20–180 min range as compared to the thrombin-stimulated cells in the absence of PIK7.

As shown in Figure 2, 100 µM PIK7 added in culture medium reduced, by about 2-fold, the basal level of the myosin regulatory light chain (RLC) phosphorylation in cultured EA.hy926 human endothelial cells and suppressed the increase in RLC phosphorylation induced by challenging cells with 100 nM thrombin. Correspondingly, 100 µM PIK7 blunted the thrombin-induced hyperpermeability of EA.hy926 cells, restoring a FITC-dextran diffusion rate observed in unstimulated EA.hy926 cell monolayers. These experiments confirmed that PIK7 retained MLCK inhibitory activity as well as the ability to cross the plasma membrane of cultured endothelial cells and reach its intracellular molecular target. Further cell-based and in vivo studies outlined the working concentrations of PIK7 as 20 µM and higher. Due to low PIK7 toxicity [17], up to millimolar concentrations of the peptide could be used in the experiments.

### 3.2. In Vivo Experiments: Effect of PIK7 and SNP on Systemic Hemodynamics

**Figure 3:**
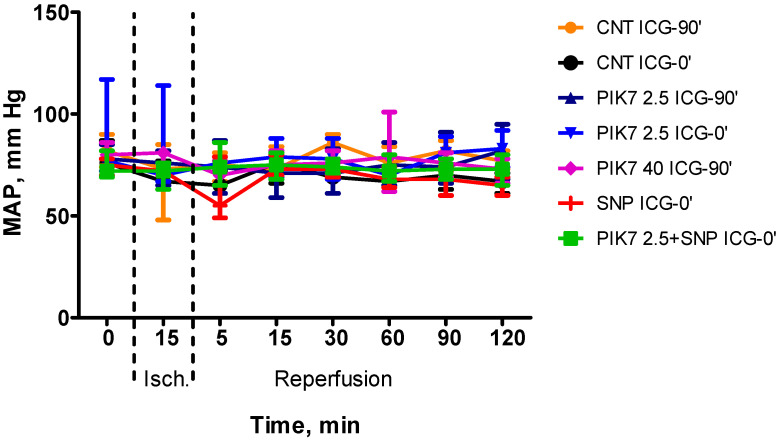
Dynamics of the level of mean arterial pressure during the experiment. Data are presented as median (Me) and interquartile range (Q1; Q3).

**Figure 4:**
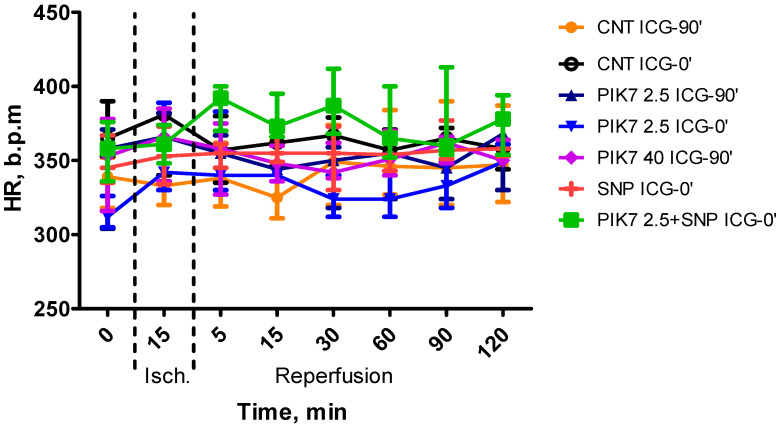
Dynamics of heart rate level during the experiment. Data are presented as median (Me) and interquartile range (Q1; Q3).

**Figure 5:**
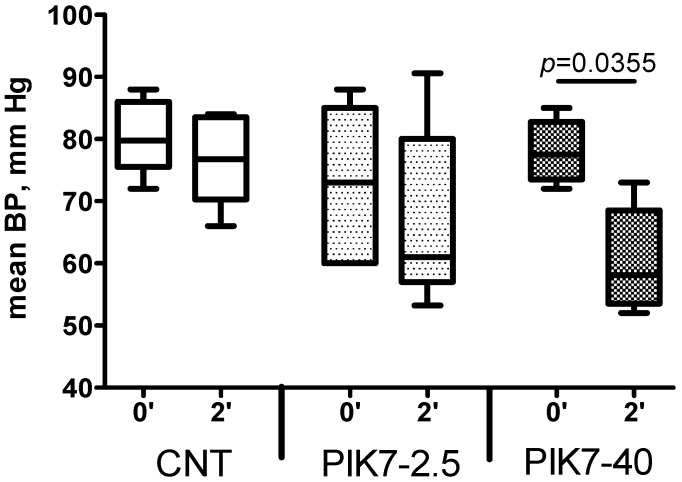
Effect of PIK7 on mean arterial pressure.

During the whole experiment, blood pressure and heart rate levels did not differ between the groups (Figure 3 and Figure 4), except for the first minutes of reperfusion, when a short-term decrease in mean arterial pressure was observed after the end of PIK7 injection. Reaching its minimum, at 1–2 min after the end of PIK7 infusion (Figure 5), mean BP gradually recovered within 3 to 5 min. This decrease was dose-dependent and resulted in a 25% [17.25–27.21] (*p* = 0.0355) decrease in mean BP in all rats in the high-dose PIK7 group (40 mg/kg) and only in three rats in the 2.5 mg/kg PIK7 group (ICG-90′).

**Figure 6:**
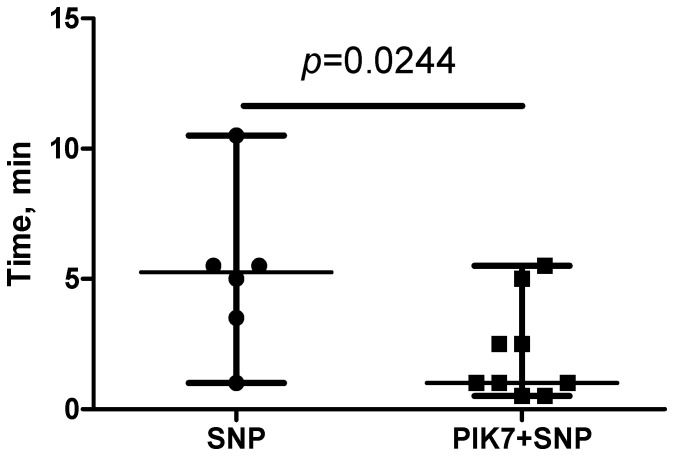
Duration of intravenous administration of sodium nitroprusside (60 μg/kg).

Although the dose (60 μg/kg) and volume (0.5 mL) of SNP were similar in the SNP and PIK7 + SNP groups, the time of SNP administration was shorter in the SNP + PIK7 group. SNP in these groups was administered intravenously and continuously at a rate to achieve and maintain a mean BP between 40 and 50 mmHg. To maintain the target BP in the PIK7 + SNP group, a higher injection rate of SNP solution was required than in the group without PIK7. Due to the increased rate of SNP administration in the PIK7 + SNP group, the time of its administration was significantly shorter than in the SNP group (Figure 6).

Although there were short-term decreases in blood pressure in the groups with PIK7 and SNP during injections of the testing substances, they did not significantly affect the size of myocardial infarction.

### 3.3. Myocardial Infarction Size

**Figure 7:**
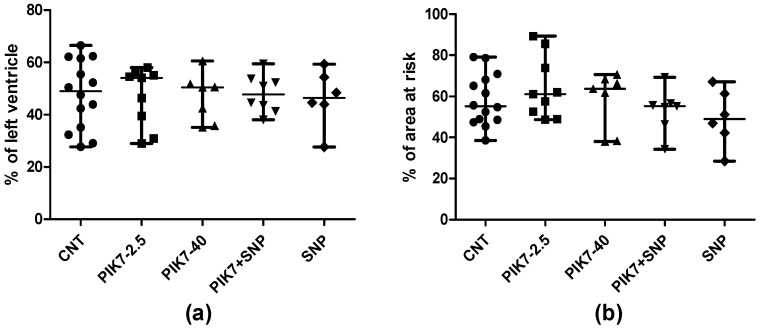
Myocardial infarction size. Sizes of area at risk (**a**) and area of necrosis (**b**).

PIK7 administration had no effect on myocardial infarction size (Figure 7). There were no significant differences in the area of risk zone between the groups. Also, there were no differences between the groups in the dynamics of the decrease in T wave amplitude at the 60th minute and 120th minute of reperfusion (Table 1). In all experiments, a significant decrease in the spectral power density of the UHR ECG was recorded in the first minute of coronary artery occlusion, which confirms the onset of myocardial ischemia. There were no differences in the spectral power density between the groups at all recording points (Table 1).

### 3.4. Size of No-Reflow Zones

**Figure 8:**
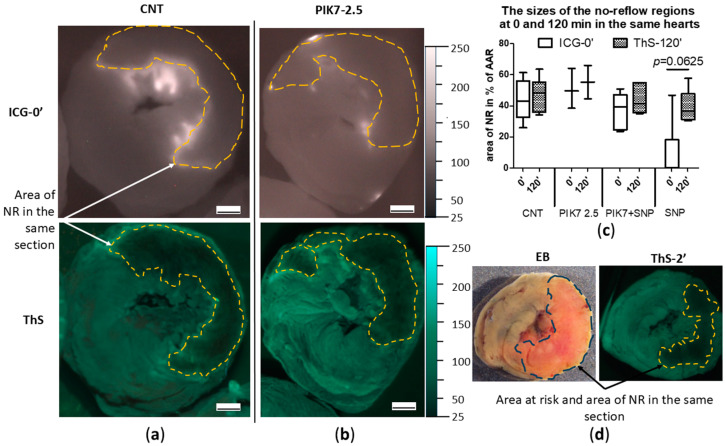
Comparison of sizes of no-reflow zones at the beginning and at the end of reperfusion in the same heart in the CNT and PIK7 2.5 groups. Representative images of ICG-fluorescence (ICG-0′) and ThS-fluorescence (ThS) in transverse sections of hearts from CNT (**a**) and PIK7 2.5 (**b**) groups. Dotted lines are the boundaries of the no-reflow (NR) zone. (**c**) Comparison of sizes of no-reflow zones obtained by planimetric analysis of fluorescence images in transverse heart slices. Comparative analysis of ICG- and ThS-fluorescence images in the SNP group revealed a trend toward an increase in the size of the no-reflow zone between the beginning of reperfusion (ICG-0′) and the end (ThS-120′). (**d**) Representative images of apical slices of the same heart stained with Evans blue: in white light (left) and in ThS-fluorescence (right). ThS was injected at the second minute of reperfusion (ThS-2′); planimetric analysis allows confirmation of the primary no-reflow observed by ICG staining. Scale bar: 1 mm.

An interesting finding of this study was the detection of early perfusion defects in the area at risk. Areas of “true” no-reflow immediately after reperfusion was detected by early ICG administration (ICG-0′), which was expressed in the absence or pronounced reduction of ICG accumulation in the intramural layer of the necrosis zone with intensive ICG retention in the subepicardial, subendocardial layers and border zones (Figure 8a–d). In representative cross-sectional images of control and PIK7 2.5 hearts, dashed lines indicate areas of primary no-reflow. To confirm the phenomenon of primary no-reflow detected using ICG, in one pilot experiment we used Thioflavin S (ThS), fluorescent vital dye staining the endothelium [11]. ThS was intravenously injected into the rat at the second minute of reperfusion after 30 min ischemia and one minute before the rat was euthanized (ThS-2′).

The results of this experiment confirmed the presence of primary no-reflow (Figure 8d). On the left side of Figure 8d is an image of a transverse apical slice of a heart stained with Evans blue (EB), where the dotted line delineates the area at risk zone, whose size in this heart was 50% of the left ventricular volume. On the right side of Figure 8d is an image of the same slice in UV light, where the dotted line outlines the zone of primary no-reflow, the size of which in this heart was 55% of the risk zone, which is comparable to the size of the necrosis zone in the control group—55% [48; 69] (*n* = 14). This indicates a pronounced blood flow disturbance in the first minutes of reperfusion in the central area of the risk zone that occupies about half of its total area. The area of the no-reflow zone measured by the ICG-fluorescence imaging method almost did not change in its size during reperfusion (Figure 8c) and was 45.7% [27.3; 53.1] in the control group in the first minutes (ICG-0′) and 44.2% [35; 54.2] at the end (ICG-90′).

**Figure 9:**
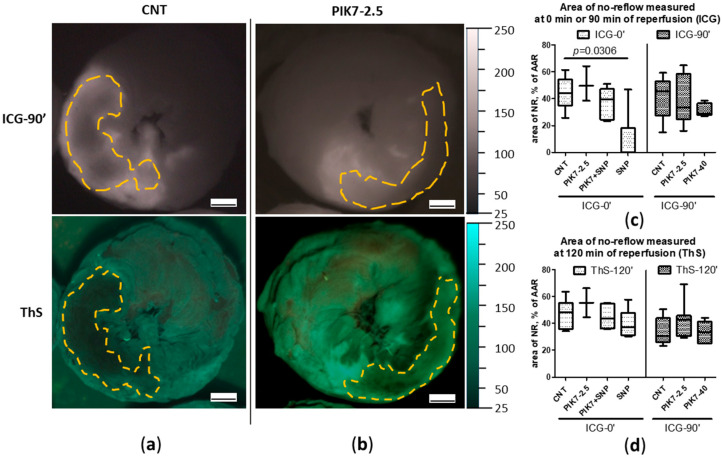
Comparison of no-reflow zone area sizes between groups with late (ICG-90′) ICG administration. Representative images of ICG-fluorescence (ICG-90′) and ThS-fluorescence (ThS) in transverse sections of hearts from CNT (**a**) and PIK7 2.5 (**b**) groups. Dotted lines are the boundaries of the no-reflow (NR) zone. (**c**) Comparison of no-reflow zone sizes at the first minutes of reperfusion (ICG-0′ subgroup) and 90 min of reperfusion (ICG-90′ subgroup) obtained by ICG-fluorescence image analysis. (**d**) Comparison of no-reflow zone sizes at the end of the second hour of reperfusion (ThS) obtained by analyzing ThS-fluorescence images. Scale bar: 1 mm.

PIK7 administration did not affect the size of both the primary and delayed no-reflow areas (Figure 8c and Figure 9a–d).

According to the original study plan, rats from the PIK7 group with early administration of ICG (ICG-0′) were to be compared with the control group (CNT, ICG-0′). However, early analysis of the data obtained at the beginning of the study revealed that early no-reflow occupies 4/5–5/6 of the necrosis zone. We decided to change the original study plan and introduce two additional groups with sodium nitroprusside (SNP and SNP + PIK7) using rats from the PIK7 2.5 ICG-0′ group, leaving only three rats, due to the redistribution of rats into two new groups.

Intravenous administration of sodium nitroprusside (SNP) during the first minutes of reperfusion resulted in a reduction or disappearance of the boundaries of the primary no-reflow zone recorded during early ICG administration (no-reflow area = 0% [0; 18]) (Figure 9c), whereas its co-administration with PIK7 at a dose of 2.5 mg/kg (PIK7 + SNP) abolished this effect of SNP on the size of the primary no-reflow area, 39.4% [24.4; 47.2] (Figure 9c).

After 2 h of reperfusion, the size of the no-reflow zones did not differ between groups (Figure 8c and Figure 9c).

### 3.5. ICG- and ThS-Fluorescence Intensity

**Figure 10:**
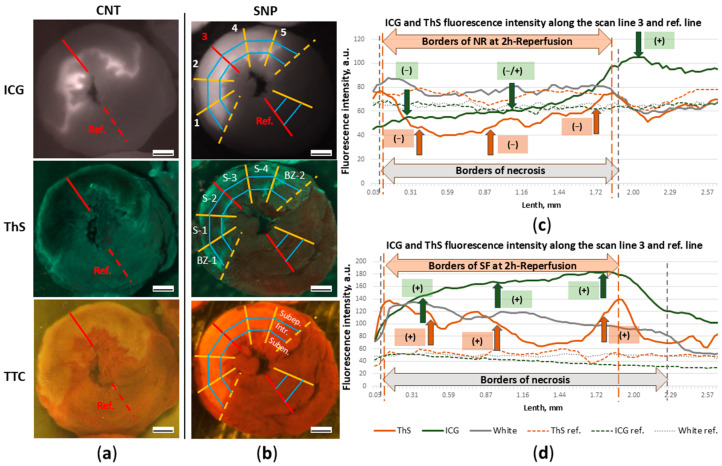
Effect of sodium nitroprusside administered during the first minutes of reperfusion on ICG-0′ fluorescence intensity in the myocardial infarction zone. Representative images of ICG-fluorescence (ICG-0′) and ThS-fluorescence (ThS) in transverse slices of hearts from CNT (**a**) and SNP (**b**) groups. (**b**) The area between the dashed lines bounding the anatomical risk zone is divided into 6 equal sectors: two border sectors (BZ-1 and BZ-2) and four inner sectors (S1–S4). Each sector is divided into three grid cells: (1) subepicardial (Subep.), (2) intramural (Intr.), and (3) subendocardial (Suben.). Ref. is the red reference line in the reference sector plotted at an equal distance from the border sectors. (**c**) and (**d**) ICG- and ThS-fluorescence intensity plots along scan line 3 from epicardium to endocardium and reference lines from CNT (**a**) and SNP (**b**) slice images, respectively. The gray “White” line is drawn from TTC-stained slice images. The three arrows in the two graphs indicate the fluorescence intensity levels (ICG or ThS) in the three myocardial layers and the “+” or “−” signs indicate the ratio to the reference fluorescence intensity level (ICG ref. or ThS ref.). NR—no-reflow, SF—slow-flow. Scale bar: 1 mm.

The data obtained in the analysis of the intensity of ICG-fluorescence enable us to estimate the severity of the endothelial barrier disfunction during reperfusion of myocardium. Figure 10a,b show images of transverse apical slices of hearts in the light of ICG- and ThS-fluorescence, from which graphs of the ICG- and ThS-fluorescence intensity were drawn along the central scanning lines (3 scan-line) from the epicardium to the endocardium and reference lines (Figure 10c,d). The graphs allow us to compare the distribution pattern of ICG-0′ and ThS in different layers of injured myocardium in the cross section of the left ventricular wall and fluorescence intensity of ICG-0′ and ThS in the control heart and the heart with SNP. By the three-fold enhancement of ICG-fluorescence intensity (green line) compared with the reference level (dashed green line), it is evident that SNP significantly enhanced fluorophore accumulation during the first minutes of reperfusion. Intensification of ICG-fluorescence intensity against the reference-line (the background) of SNP action is marked on the graph by green arrows with positive “+” signs (positive contrast). In the control heart in the subepicardial layer of the injured zone, ICG accumulation is lower than the reference level and is marked by the negative sign “−” (negative contrast). In the intramural layer, the level of ICG-fluorescence fluctuates between negative and positive contrast compared to the reference one, which indicates a low level of ICG accumulation in these layers of the damaged zone.

After two hours of reperfusion in the SNP rat, despite the increased vascular permeability in the injury zone during the first minutes of reperfusion, the intensity of ThS-fluorescence is higher (orange line) than in the reference zone (Figure 10d). In the control experiment, in the same zone, the intensity of ThS-fluorescence is lower than in the reference zone (Figure 10c). Consequently, the SNP-induced short-term increase in blood flow in the injury zone, followed by an increase in ICG deposition in regions of overt endothelial disruption at the beginning of reperfusion, does not reduce blood flow after two hours in the necrosis zone, leading to slow flow (SF) in infarct zone in some rats instead of no-reflow (Figure 10d).

**Figure 11:**
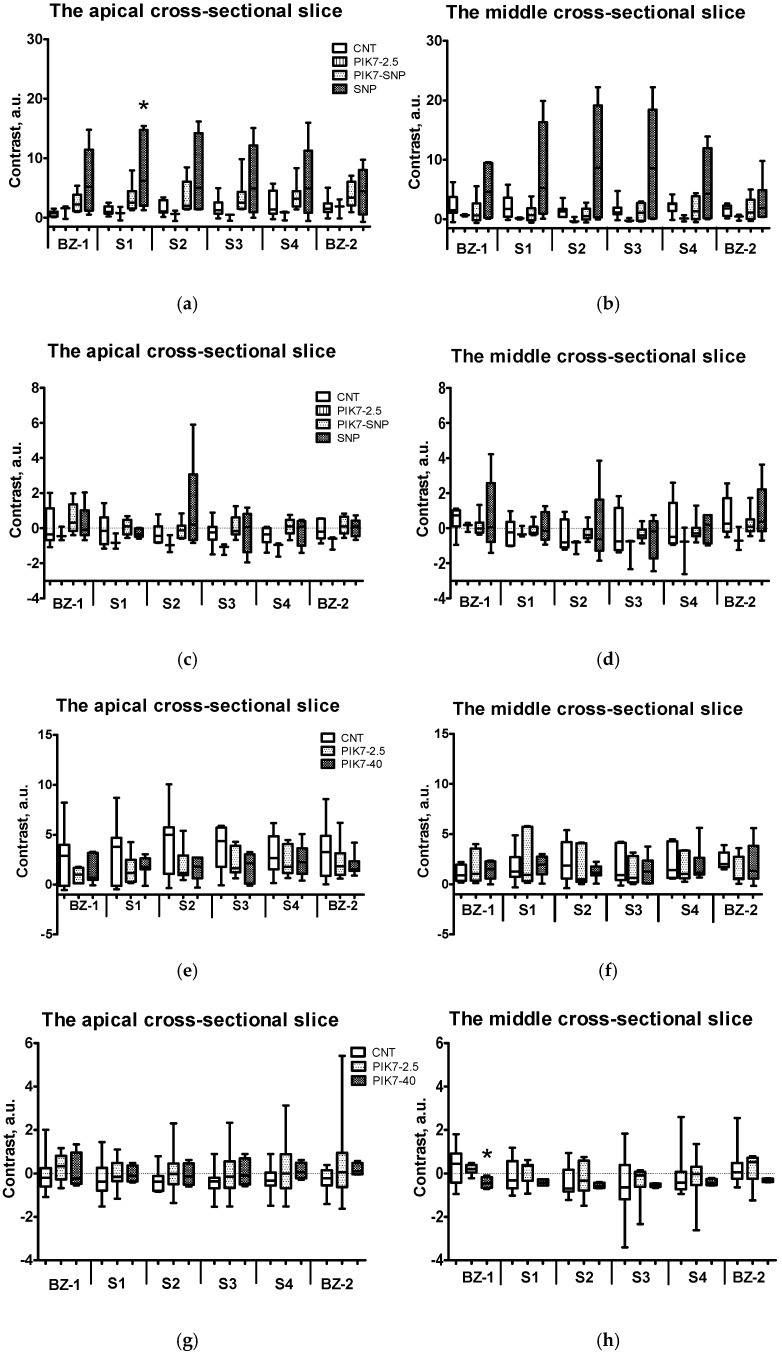
Comparison of contrast between sectors of the intramural layer of the left ventricular wall in the risk zone and the remote zone (interventricular septum) at different time points. (**a**,**b**) ICG-fluorescence intensity in the intramural layer of apical (**a**) and midline (**b**) slices in groups with early ICG administration (ICG-0′). (**c**,**d**) Intensity of ThS-fluorescence in the intramural layer of apical (**c**) and medial (**d**) slices in groups with early ICG administration (ICG-0′). (**e**,**f**) ICG-fluorescence intensity in the intramural layer of apical (**e**) and midline (**f**) slices in groups with delayed administration of ICG (ICG-90′). (**g**,**h**) Intensity of ThS-fluorescence in the intramural layer of apical (**g**) and medial (**h**) slices in groups with late ICG administration (ICG-90′). BZ-1 and BZ-2 are border sectors of the risk zone (Figure 9b); S1, S2, S3, and S4 are inner sectors. *—statistically significant difference (*p* < 0.05) with the same cell in the control group.

To evaluate the effect of PIK7 on vascular permeability and the severity of microvascular obstruction, we compared the intensity of ICG- and ThS-fluorescence in the risk zone in each grid cell (Figure 10b) of each heart in apical and middle slices (Figure 11a–c). To compare the fluorescence intensities of the two fluorophores, we used the parameter “contrast”, which reflects the ratio between the fluorescence in the examined (injured) myocardial zone and the reference (remote) zone, which was calculated using a formula after subtracting the background fluorescence values (see Materials and Methods). The contrast value is directly proportional to the fluorescence intensity in the ROI, and its negative values occur when the fluorescence intensity of the ROI decreases below the fluorescence intensity of the reference zone.

### 3.6. Comparison of Contrast of ICG-Fluorescence Intensity Between Sectors in the Intramural Layer of ICG-0′ Groups

Comparison of contrast between the border and central sectors in the case of early ICG administration showed no differences in all groups. There were also no contrast differences between the PIK7 2.5 groups and the control group. ICG-fluorescence intensity levels were higher in the SNP group compared with the control group, particularly in the first inner sector (S1) in the subepicardial (4.86 a.u. [1.46; 18.90] vs. 0.67 a.u. [0.17; 0.97]) and intramural (6.15 a.u. [2.00; 14.76] vs. 0.97 a.u. [0.67; 1.91]) layers (Figure 11a). This indicates that SNP-induced enhancement of coronary blood flow in the entire risk zone, including the primary no-reflow area, resulted in pronounced hyperpermeability, a significant increase in ICG accumulation in the intramural layer, leading to an increase in its fluorescence intensity, especially in the S1 sector, and a decrease in the primary no-reflow area (Figure 8c). Figure 11c,d show higher ThS-fluorescence contrast value in the central sector of the apical slice and in the central and border sectors in the middle slice, although these differences were not statistically significant. This suggests that, in some rats, blood flow in the intramural layer and border sectors was preserved (slow flow) in the second hour of reperfusion, and there was increased vascular permeability in these areas. Fluorescence peaks at the edges of the necrotic zone indicate preserved blood flow and increased fluorophore accumulation.

Intravenous co-administration of SNP with PIK7 at a dose of 2.5 mg/kg partially reversed the hemodynamic effect of SNP at a dose of 60 µg/kg (Figure 5 and Figure 6), accompanied by a two- to three-fold decrease in ICG-0′ fluorescence intensity in all sectors, compared with the SNP group (Figure 11a); there were also no differences in the size of the primary no-reflow area between the SNP + PIK7 and CNT groups (Figure 9c).

Analysis of ThS-fluorescence in the PIK7 40 group revealed a slight decrease in contrast in the mid-slice at the edges of the no-reflow zone and a significant decrease in contrast in the first border zone (BZ-1) to −0.45 a.u. [−0.65; −0.18] vs. 0.44 a.u. [−0.43; 0.92] in the control group (Figure 9d), whereas comparison of the fluorescence intensity of ICG administered at 90 min (ICG-90′) showed no significant differences between the same groups of rats.

### 3.7. The Severity of Blood Stasis

The absence of significant differences in the severity of microvascular obstruction is confirmed by the results of planimetric analysis of histologic transverse sections of hearts stained with Mallory trichrome. Thus, the area occupied by erythrocytes (stasis) in the control group was 11.1% [5.2; 13.6], and it was 9.7% [6.1; 11.9] and 10.1% [2.2; 13.8] in the PIK7 2.5 and PIK7 40 groups, respectively. In the two SNP groups, the area occupied by erythrocytes was also comparable to the control group: 12.3% [9.3; 15.4] in the SNP group and 14.6% [8.8; 17.8] in the SNP + PIK7 group. The area occupied by red blood cells in the remote zone did not exceed one percent.

### 3.8. Electron Microscopy Data

**Figure 12:**
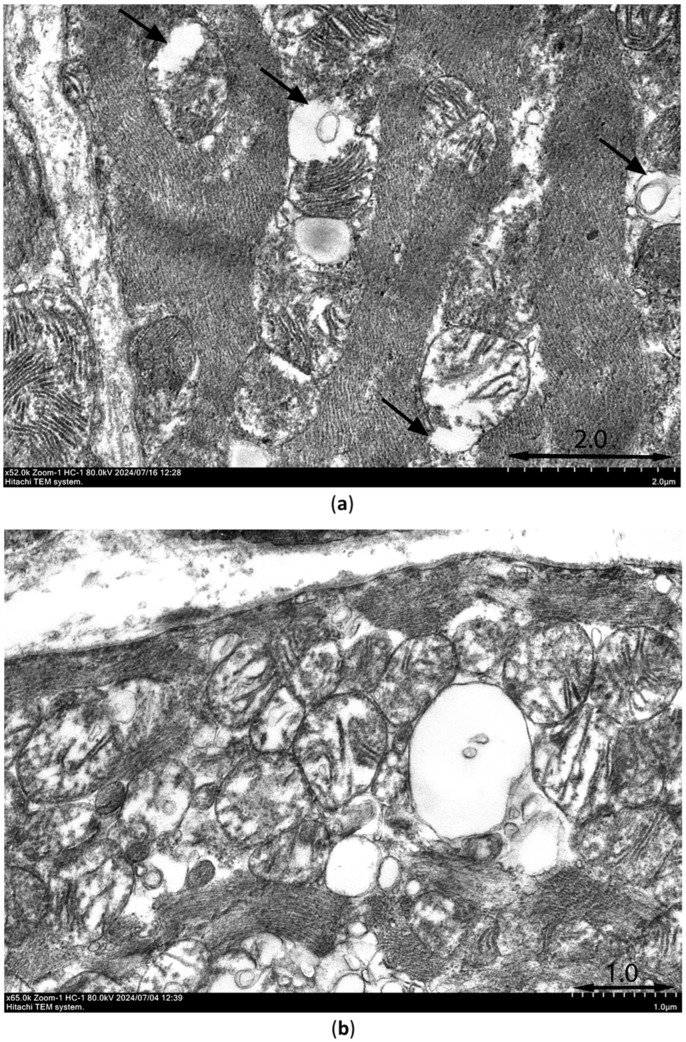

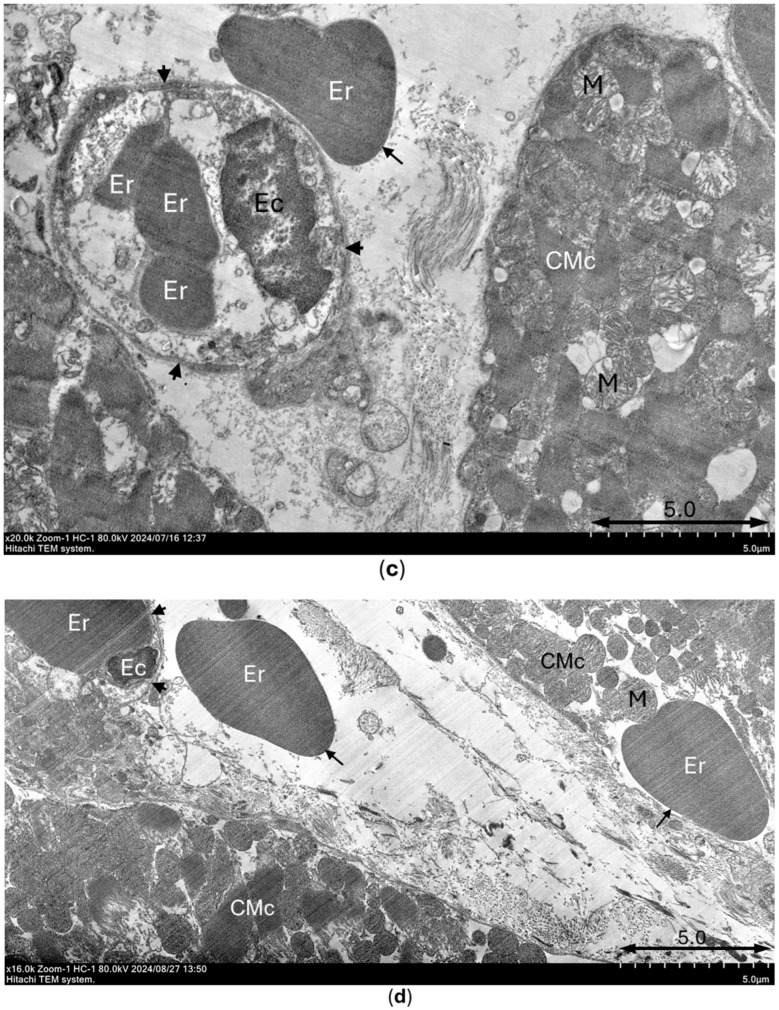

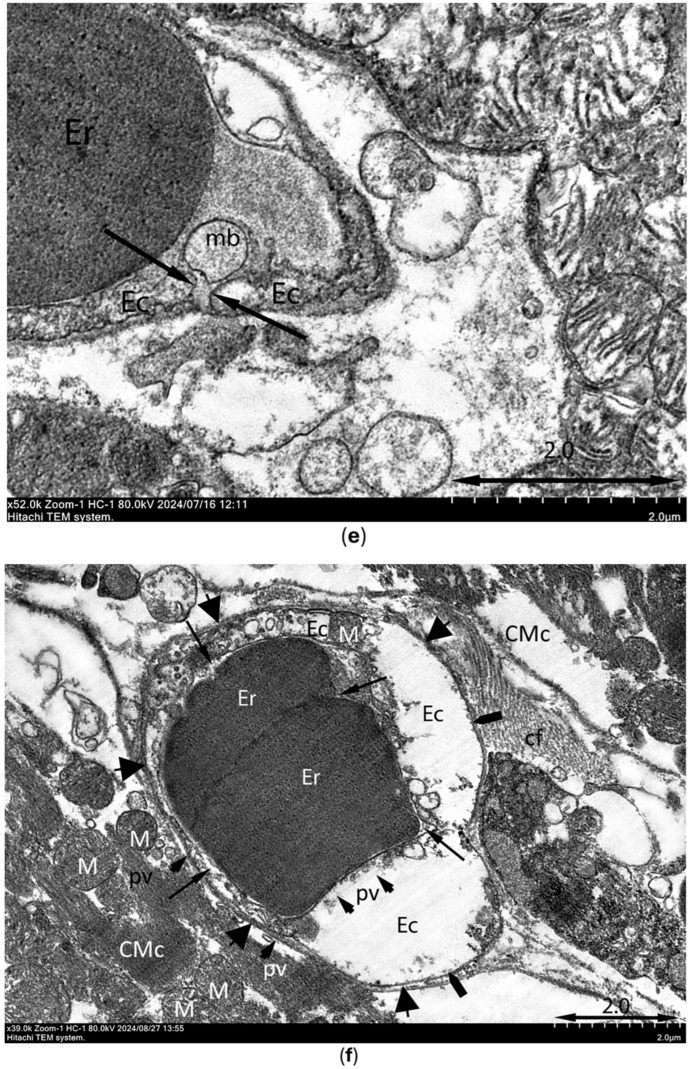
Representative electronograms of myocardial samples taken from the intramural layer of the central sectors of the zone at risk from control rats (**a**,**c**,**e**) and PIK7 2.5 rats (**b**,**d**,**f**) at 10 min of reperfusion. (**a**) Black arrows show mitochondria with rupture of the outer membrane. (**c**,**d**) Arrows point to the extravasated erythrocyte; short thick arrows—basement membrane. (**e**) Arrows point to the open interendothelial space with a membrane bleb (mb) adjacent to it. (**f**) Two pentagonal arrows point to protrusions of edematous endothelium into the capillary lumen containing two erythrocytes: Ec—endotheliocyte; thin arrow—capillary lumen (the lumen of a capillary in which two erythrocytes are stuck together); Er—erythrocytes; PV—pinocytic vesicles, short arrows; short thick arrows—basement membrane; M—mitochondria; cf—collagen fibers; CMc—cardiomyocyte. Electronograms were taken with a transmission electron microscope HITACHI7800 at magnifications of 52,000 (**a**), 65,000 (**b**), 20,000 (**c**), 16,000 (**d**), 52,000 (**e**) and 39,000 (**f**).

Ultrastructural signs of ischemia/reperfusion injury are shown in Figure 12. Transmission electron microscopy was used to observe samples of the intramural layer of the necrosis zones after 30 min ischemia and 10 min reperfusion in control rats and rats injected with PIK7 2.5 mg/kg. Signs of irreversible damage of cardiomyocytes were visible in samples from rats with PIK7 2.5 and were obvious in the control rats. In samples from the control rats, we found marked mitochondrial edema, matrix flakes, cristae separation, and ruptures of the outer mitochondrial membrane; in addition, vacuolization (Figure 12a), intercellular edema, and lipid granules were detected, indicating irreversible damage to the cardiomyocyte. The same signs of irreversible damage were seen in almost all cardiomyocytes in the sample from the PIK7-2.5 heart; however, mild to moderate mitochondrial swelling was more commonly observed in the experimental sample (Figure 12b). The mean mitochondrial area of the experimental sample was 0.71 ± 0.02 μm^2^ (*n* = 145), which was half that of the control group: 1.43 ± 0.06 μm^2^ (*n* = 282).

Although we found a few erythrocytes outside the capillaries (extravasation) in the control and experimental samples (Figure 12c,d), there were a few cases of gap formation with separation of endotheliocytes (Figure 12e) in the control group and none in the experimental sample. At the same time, there were signs of no-reflow phenomenon in two samples, such as a severe degree of endotheliocyte edema leading to complete obturation of the capillary lumen by membrane bulges (protrusions, Figure 12f) and membrane-bound blebs extending into the capillary lumen. A quarter of capillaries in the control sample had normal outlines and isolated cases of spasm, and more than half of the capillaries had blood cells in the lumen. In contrast to the control sample in the experimental sample, one third of capillaries had narrowed lumen due to more active tone.

## 4. Discussion

Ultrastructural alterations of the microvasculature caused by ischemia-reperfusion injury begin to form during ischemia [12]. Experiments on dogs have shown that prolonged ischemia causes intracellular edema, with the appearance of membrane-bound blebs on the luminal surface of endotheliocytes, which partially or completely obturate the capillary lumen [9,12,29]. The initial area of their appearance during ischemia is the center of the risk zone with low levels of residual blood flow and oxygen. When blood flow is restored in such areas, flow slows down or is not restored due to the development of primary (early) no-reflow [9]. Obviously, it is possible to prevent or reduce the manifestations of primary no-reflow by timely reperfusion. The second stage of edema formation starts with reperfusion and is associated with myocardial reperfusion injury, during which intracellular edema increases, and interstitial edema begins to form [9,30]. The interstitial edema is driven by two processes that happen simultaneously during reperfusion: the increased vascular permeability [6,30,31] and a decrease in myocardial lymphatic outflow from the injured myocardium [32]. Impaired vascular barrier function causes microvascular leakage, which is considered as a therapeutic target for ischemia and reperfusion injury [33]. Reperfusion-induced interendothelial cellular gap formation is mediated by increasing MLCK activity [14,18,34]. This fact prompted us to study the effectiveness of a new MLCK inhibitor, PIK7 [17], in a model of myocardial infarction in rats to evaluate its microvascular hyperpermeability counteracting effect and its capacity to limit no-reflow conditions.

At first, using an in vitro model to study the endothelial barrier function and paracellular permeability, we provided experimental evidence that PIK7 inhibits MLCK activity in cultured endothelial cells, and this inhibition results in the blocking of endothelial hyperpermeability induced by a potent edemagenic agent, thrombin. These results repeat the previous data presented in [17]. To test endothelial barrier function and paracellular permeability in vivo, we chose the ICG imaging technique. We believe that ICG imaging is best suited for this purpose, since the half-life of the ICG fluorophore is short, and the effect of PIK7 at different time points in an acute experiment is best estimated using the ICG imaging method. In its classical form, the Miles test for increasing vascular permeability uses Evans blue, which has a circulation time of hours rather than minutes, as in indocyanine green [35].

For the first time, using early ICG administration, we have detected early no-reflow in rats after 30 min regional myocardial ischemia. In the circulation, ICG binds to albumin and high- and low-density lipoproteins, allowing its use as a marker of increased vascular permeability, since intact vascular endothelium is “impermeable” to albumin [13,35]. ICG-fluorescence images of control rats show that injection during the first minutes of reperfusion ICG accumulates only at the edges of the necrosis zone, where its maximum fluorescence intensity is observed. This indicates the highest vascular permeability at the edges of the necrosis zone, while the center of the necrosis zone in the intramural layer remains dark relative to the edges. Insufficient intensity of ICG-fluorescence in the center of the necrosis zone can be explained both by the absence of blood flow in this area and by the absence of increased vascular permeability in the center of the necrosis zone in conditions of low oxygen content and activators of endotheliocyte contraction triggering paracellular transport. Electron microscopy data of myocardial samples taken from the center of the necrosis zone of control and experimental rats at 10 min of reperfusion confirm the assumptions made based on ICG-fluorescence image analysis. In the center of the intramural layer of the risk zone, membrane-bound blebs within the capillary lumen were detected, which partially or completely obturated the lumen. This is likely related to endotheliocytes swelling during ischemia due to ionic transport dysregulation. In a few fields of view, there was erythrocyte diapedesis, and in the control rat there were only single cases of endothelial intercellular gap formation.

Earlier in experiments in rats with 30 min ischemia, increased vascular permeability was observed throughout the necrosis area, with a maximum in its center, while early no-reflow was not detected both in vivo [21,36,37,38] and in experiments on the isolated heart [39] in the presence of signs of irreversible damage to cardiomyocytes in the risk zone [39]. Argano et al. (1996) reported the occurrence of no-reflow in the isolated blood-perfused rat heart model after 30 min ischemia and 40–80 min reperfusion and demonstrated the efficacy of SNP in its treatment [25]. In other animal species, early no-reflow is also not pronounced or is absent after 30 min ischemia. For example, in rabbits, early no-reflow only begins to appear (12% of the risk zone) after 30 min myocardial ischemia [29], and in dogs only after 90 min ischemia [9]. The appearance of early no-reflow in the present study can be explained by the unusually low tolerance to ischemia of rat myocardial endotheliocytes. We did not change the experimental conditions in the present study. There are only two differences known to us from previous studies, which are the source of rats and the age of the rats (16 weeks), which were 4 weeks older than the previous rats. We found no unusual electrocardiographic measurements with either conventional or ultra-high-resolution ECG.

Reduced tolerance of endotheliocytes to ischemia was manifested in the appearance of early microvascular obstruction after a relatively short period of ischemia—30 min. Intracellular edema covered the entire intramural layer and adjacent subendocardial and subepicardial regions in such a way that, by the end of 30 min, ischemia the size of the no-reflow zone occupied 40% of the risk zone. A leading role in the mechanism of early no-reflow in rats in this study is likely played by endothelial blebbing, because we found no evidence of significant vasoconstriction or external compression of capillaries in myocardial samples from control rats at 10 min of reperfusion. It is possible that blebbing occurs as a defense mechanism to prevent endothelial cell necrosis when the usual ways of cell repair are no longer effective [40]. Also, there is evidence that supports the version of intracellular edema, rather than the formation of the final stage of apoptosis, because the final stage of apoptosis occurs only during the reperfusion phase [41]. Nevertheless, the absence of enlargement of no-reflow area between the beginning and the end of reperfusion lead us to conclude that injury of endothelial cells in one third of the entire risk zone occurred during ischemia. In other words, the comparison of no-reflow zone sizes obtained by ICG-0′ fluorescence images did not significantly differ from the sizes of the no-reflow areas obtained by ThS-fluorescence images in the same rats in all groups except the SNP group. By the end of two-hour reperfusion, the size of no-reflow zones was 30–40% of the area of the risk zone, which is comparable to previous findings [42].

The basic idea behind the inhibition of MLCK at the beginning of post-ischemic hyperemia is the reduction of no-reflow severity due to the prevention of endothelial hyperpermeability and myocardial edema progression. This idea is supported by evidence of the protective effect of MLCK inhibition in in vitro [14,43] and in vivo experiments [44]. The genetic deficiency of 210 kDa MLCK in mice, as well as MLCK inhibition by a small-molecule organic inhibitor, increases resistance to lung injury in sepsis models by preserving the endothelial barrier function [16]. In the present study, peptide inhibitor PIK7 was used for the inhibition of MLCK in vivo [17]. We anticipated that, after a single administration of PIK7 at the very beginning of reperfusion, vascular permeability and intercellular edema in the risk zone would be reduced, which would reduce microvessel compression and ultimately improve microcirculation in injured myocardium.

Bolus intravenous administration of PIK7 just before the beginning of blood flow restoration decreased the intensity of ICG-0′ fluorescence (the level of contrast between the investigated zone and the reference zone), but not significantly due to the small number of observations (*n* = 3). Electron microscopy of samples of intramural myocardium obtained at the 10th minute after injection of PIK7 2.5 mg/kg showed the anti-edema effect of PIK7 compared to the heart samples from the control group. Also, the samples from experimental animals showed an increase in the number of vessels in a state of pronounced tone compared to the control animal (40.7% and 7.5%, respectively). Interestingly, in the intramural layer in control and experimental rats there were almost no cases of weakening of intercellular contacts in capillaries. This suggests that, in the center of the early no-reflow zone, the endothelial barrier is preserved 10 min after the beginning of reperfusion due to the reduced activation of endotheliocytes in conditions of insufficient blood flow. However, signs of short-term post-ischemic hyperemia were observed in the control sample, accompanied by increased vascular permeability in the intramural layer, namely isolated cases of erythrocyte diapedesis. The consequences of which, in the form of increased intra- and intercellular edema, were reduced due to the inhibition of MLCK in groups with PIK7 in the first minutes of reperfusion.

The anti-edema effect of PIK7 was transient, as revealed by ICG administration, at 90 min of reperfusion and comparison of ICG-fluorescence intensity in the necrosis zone between the three groups: CNT, PIK7 2.5, and PIK7 40. The levels of ICG accumulation did not differ between the groups.

To evaluate the efficacy of PIK7 under the conditions of vasodilator action used to treat primary no-reflow, PIK7 and SNP were administered simultaneously via different intravenous catheters. Co-injection of PIK7 solutions with SNP significantly shortened the infusion time of SNP compared with SNP without PIK7. The effect of PIK7 on the hypotensive effect of SNP allows us to consider PIK7 as a potential drug for the treatment of refractory vasoplegia.

The NO donor SNP eliminated primary no-reflow, but at the same time increased ICG-0′ fluorescence intensity of the necrosis zone, indicating an increase in vascular permeability over the entire area of the necrosis zone. Co-injection of PIK7 with SNP significantly reduced vascular permeability, but the area of primary no-reflow decreased not as significantly as in the SNP group. By the end of two-hour reperfusion, blood stasis was formed, the severity of which did not differ between the groups, and the infiltration of the intramural layer by segmented neutrophils (data not presented) was maximal in the PIK7-40 and SNP groups and minimal in the PIK7 + SNP group.

## 5. Conclusions

In the present study, a single intravenous administration of MLCK inhibitor, PIK7, was found to reduce vascular hyperpermeability caused by ischemic and reperfusion injury. The effect of PIK7 at a dose of 2.5 mg/kg is transient and ceases within 90 min of reperfusion, so it is reasonable to evaluate its antiedemic effect with repeated administration or prolonged infusion. The early no-reflow detected for the first time in rats after 30 min ischemia reduces the area accessible for PIK7 action. Co-injection of PIK7 with SNP increases blood flow in the early no-reflow zone while reducing the increased vascular permeability caused by SNP. The interaction of SNP and PIK7 in vascular permeability requires further study to provide useful information for improving therapeutic strategies.

One more observation should be mentioned, which refers to the absence of significant differences in the areas of primary and secondary no-reflow zones, indicating more significant ischemic myocardial damage and an insignificant role of reperfusion damage in rats with low myocardial tolerance to ischemia. The absence of increased vascular permeability in areas with severe intracellular edema indicates the absence of an obvious relationship between the mechanisms causing them. The severity of intracellular edema of the ischemic myocardium depends on the tolerance of the myocardium to ischemia and is directly proportional to its duration, while increased permeability manifests itself in the first minutes of reperfusion and requires a certain level of blood flow and generation of reactive oxygen species. In the area of “true” (early) no-reflow phenomenon, blood flow is almost absent, which reduces the manifestations of increased permeability.

There are opinions that microcirculatory damage in the areas at risk and no-reflow can increase myocardial infarct size [30]. Our result with 30 min ischemia and two-hour reperfusion, as well as measurement of the no-reflow zone after 24-h reperfusion in rats and mice, showed that the no-reflow zone is always smaller than the necrosis zone and is a consequence of irreversible myocardial damage rather than its cause [29].

## Figures and Tables

**Table 1:** Validation of left coronary artery ligation and reperfusion by using electrocardiography.

	The Spectral Power Density of the UHR ECG, mW/Hz		The ST Segment Resolution of Conventional ECG, %	
Groups	Baseline	1st min of Ischemia	60th min of Reperfusion	120th min of Reperfusion
CNT	4.89 [3.57; 5.91]	3.27 [2.31; 4.34] *	57.8 [34.2; 74.1]	68.5 [42.0; 72.5]
PIK7_2.5_	4.81 [3.66; 5.87]	3.45 [2.33; 4.21] *	74.3 [58.4; 97.5]	55.7 [36.16; 84.4]
PIK7_40_	5.01 [3.72; 6.21]	3.34 [2.40; 4.33] *	35.4 [−133.9; 74.1]	38.4 [−165.9; 76.5]
PIK7_2.5_ + SNP	4.87 [3.63; 5.97]	3.38 [2.42; 4.31] *	79.2 [22.5; 153.3]	60.5 [27.7; 97.9]
SNP	4.94 [3.68; 6.02]	3.31 [2.38; 4.41] *	51.3 [20.2; 82.8]	51.4 [5.5; 69.6]

* indicates *p* < 0.05 when data are compared to those for the baseline.

## Data Availability

Data underlying the results presented in this paper are not publicly available at this time but may be obtained from the authors upon reasonable request.
